# A scoping review of the impact of transverse carpal ligament sectioning on the thumb carpometacarpal joint: Does it increase the risk of ostearthritis?

**DOI:** 10.1016/j.jpra.2026.01.029

**Published:** 2026-01-23

**Authors:** Quinn Steiner, Robert George, Armin Edalatpour, Pradeep K. Attaluri, Brett F. Michelotti

**Affiliations:** Division of Plastic and Reconstructive Surgery, Department of Surgery, University of Wisconsin - Madison, Madison, WI, USA

**Keywords:** Carpal tunnel release, Carpometacarpal joint, Transverse carpal ligament, Osteoarthritis

## Abstract

**Purpose:**

We aimed to review the effects of carpal tunnel ligament sectioning on the thumb carpometacarpal (CMC) joint with the hypothesis that carpal tunnel release (CTR) may result in biomechanical changes at the thumb CMC joint predisposing an individual to pain and or accelerated articular wear.

**Methods:**

This review was performed using the Pubmed and Medline databases from January 1, 1970 to January 7, 2023. Articles that examine the thumb CMC joint after CTR were categorized as cadaveric, clinical, or computational studies. The anatomy, pathophysiology, and treatment of both CMC arthritis and carpal tunnel syndrome (CTS) were included to provide context for our results. These studies were synthesized to describe the effects of CTR on the thumb CMC joint.

**Results:**

The search resulted in 1093 articles for possible inclusion. After screening abstracts and removing duplicates, 48 articles were included in this review. Common themes from this review include: widening and differences in configuration of the carpal arch following CTR, and changes in the positioning and movement of the carpal bones—leading to differences in the forces and contact stressors across articulations. We also review how these changes may lead to thumb CMC pain and could contribute to articular wear.

**Conclusion:**

There are morphologic and biomechanical changes that occur across the carpal arch after CTR. These changes may be associated with differences in intrinsic hand muscle function, increased ligamentous stress, and accelerated ligamentous laxity - leading to alterations in the distribution of force around the trapezium and to its articular cartilage.

## Introduction

Thumb basal joint arthritis or carpometacarpal arthritis (CMC) has been reported in 8%–12% of the general population with the prevalence of symptomatic disease between 3% and 5%.^1^ Pain associated with this condition can be debilitating, considering the utility of the thumb in everyday activities.

Carpal tunnel syndrome (CTS) is the most common and widely studied compressive neuropathy.^2^ CTS symptoms vary and include pain, paresthesia, and weakness that can be interfere with patients’ quality of life.^3^

Carpal tunnel syndrome often coexists with basal joint arthritis and this association has been acknowledged by previous studies.^4^, ^5^, ^6^ It has also been noted that in patients undergoing basal joint arthroplasty, up to 42% of them, have had a prior carpal tunnel release (CTR).^5^ Possible etiologies for these concomitant pathologies have been proposed. One thought is that these two entities may occur together simply as a product of their prevalence in the general population. Another postulation is that in certain populations, for example patients with rheumatoid arthritis and gout, there may be coexistent synovitis of the basal joint and flexor tendons leading to concomitant pathology. These hypotheses, and prior research, also indicate that there may be a relationship between the biomechanics of the thumb CMC joint and the transverse carpal ligament.

We aim to review the effects of transverse carpal ligament sectioning on the thumb CMC joint with the hypothesis that CTR may result in biomechanical changes at the thumb CMC joint predisposing an individual to pain and/or accelerated articular wear.

## Methods

This review was performed using the Pubmed and Medline databases from January 1, 1970 to January 7, 2023. A list of search terms used can be found in Supplemental Table 1.

The search resulted in 1093 articles for possible inclusion. After screening abstracts and removing duplicates, 48 articles were included in this review. Articles that examine the thumb CMC joint after CTR were categorized as cadaveric, clinical, or computational studies. The anatomy, pathophysiology, and treatment of both CMC arthritis and CTS were included to provide context for our results. These studies were synthesized to describe the effects of CTR on the thumb CMC joint.

## Results

### CMC background

#### Anatomy

The basal joint of the thumb consists of the articulation between the base of the thumb metacarpal and the trapezium. The movements about the CMC joint include antepulsion, retropulsion, abduction, adduction, pronation, supination, and opposition. The reciprocal surfaces of the trapezium and metacarpal base create a biconcave, saddle-shaped joint that permit movements in multiple planes.^7^^,^^8^ The articular surface of the first metacarpal is significantly larger than the articular surface of the trapezium and the outer ridges guide the antepulsion and retropulsion movements. The concavity of each articular surface is shallow and the joint itself has little intrinsic stability. Therefore, the primary stability of the joint is afforded by the surrounding ligaments.^8^^,^^9^

There are many ligaments that stabilize this joint, however several have been described as the primary stabilizers. The dorsal radial ligament (DRL), which extends between the dorsal tubercle of the trapezium and the base of the first metacarpal, restricts abduction of the thumb. The posterior oblique ligament (POL) attaches the medial surface of the first metacarpal to the medial trapezium and constrains supination. The dorsal ligament complex includes both the DRL and the POL, which is thick and short relative to the thinner anterior oblique ligament (AOL), commonly named volar beak ligament. The AOL also arises from the volar trapezium to insert on the base of the thumb metacarpal. It serves to resist abduction, extension, and pronation. Lastly, the intermetacarpal ligament (IML) connects the proximal first and second metacarpals and restricts abduction.^9^, ^10^, ^11^, ^12^

#### Pathophysiology of CMC arthritis

Basal thumb arthritis is so prevalent in the general population it is considered by some to be a result of normal aging.^13^ It is thought to result from ligamentous degeneration of the stabilizers of the CMC joint. This ligamentous laxity permits movement of the metacarpal base across the trapezium which produces significant shear forces within the volar compartment of the joint. This shear is exacerbated with recurring stress to the CMC joint.^14^^,^^15^ A decrease in the strength of the AOL, IML, and POL destabilizes the CMC joint, increasing risk of subluxation.^9^^,^^11^^,^^12^ This repeated loading of a subluxed joint leads to articular cartilage damage. Some authors speculate that CTS may contribute to the risk of joint subluxation via neuropathy-mediated motor dysfunction of the intrinsic hand muscles. Li et al. demonstrated that patients with CTS, even without thenar atrophy, demonstrate disrupted thumb opposition and circumduction despite normal pinch strength.^16^

#### Treatment of CMC arthritis

Non-surgical options for CMC osteoarthritis (OA) include hand therapy, non-steroidal anti-inflammatory medications, corticosteroid injections, and autologous fat and platelet-rich plasma injections. Previous analysis has demonstrated increased cost effectiveness of steroid injections compared to surgery in patients with CMC OA. Patients in the surgical group had both increased healthcare costs as well as costs associated with time off from employment at 1 year.^17^ For patients with refractory symptoms surgical management can be considered. There are numerous techniques, and options for thumb CMC pain and early OA, including thumb metacarpal osteotomy, ligament reconstruction, arthrodesis, or minimally invasive arthroscopic techniques.^18^, ^19^, ^20^ For more severe disease, trapeziectomy with or without ligament reconstruction or interposition is the reference standard.^18^^,^^14^^,^^21^

### CTS background

#### Anatomy

The carpal tunnel is defined as the space between the flexor retinaculum (FR) and the carpal bones, containing nine flexor tendons and the median nerve. The median nerve receives sensory input from the three radial digits and the radial half of the ring finger.^22^ Branches of the median nerve include the palmar sensory cutaneous branch and the recurrent motor branch.^23^^,^^24^ The volar border of the carpal tunnel is created by the fibrous transverse carpal ligament (TCL) which is the intermediate portion of the FR.^22^^,^^25^

The origins of the FR have been well described and are important to consider when discussing the carpal tunnel anatomy and biomechanics of the carpal bones. The FR begins proximally as the deep investing fascia of the flexor compartment before transitioning into thick fibrous TCL.^25^ The TCL attaches proximally at the pisiform and scaphoid and distally at the trapezium and hamate.^25^^,^^26^ The insertions of the TCL were well characterized in a study by Manley et al.^26^

The TCL also serves as the origin for several muscles. Abductor pollicis brevis (APB), the superficial belly of flexor pollicis brevis (sFPB), and the opponens pollicis (OPP) originate from the TCL. The sFPB occupies an almost negligible area on the TCL while the APB and OPP occupy nearly 20%. The OPP origin is located near the trapezium and is the main player in thumb opposition while APB origin lies away from the trapezium which permits a mechanical advantage for thumb abduction and pronation.^27^ Destabilization of these muscle origins may lead to altered thumb biomechanics.

#### Pathophysiology

There are several theories for the etiology of CTS, including compression of the median nerve leading to ischemia and disruption of the endoneurial microvessels which are important for nutrition.^28^ Other theories discuss inflammation and adhesion formation of the perineurial connective tissue decreasing mobility of the median nerve and resulting in edema which then increases the diffusion distance for oxygen, leading to hypoxia.^22^^,^^24^^,^^23^

There are two common sites for compression to occur. The first is at the proximal edge of the TCL and is the most probable site for flexion induced changes in the median nerve.^22^^,^^25^ The second is at the level of the hook of the hamate which is where the canal is narrowest and has greatest chance for median nerve compression.^22^^,^^25^ The pathophysiology of CTS offers insight as to why it has been demonstrated to be associated with pre-existing or concomitant CMC arthritis.^6^^,^^29^ For example, Crosby et al. noted in patients with basal joint OA there was a pattern of midcarpal collapse leading to decreased volume of the carpal tunnel which they speculated increased risk of CTS.^15^^,^^30^

#### Treatment of CTS

Management of CTS is based on disease severity. Splinting, physical therapy, and corticosteroid injections are commonly employed non-surgical measures.^3^ Surgical management can also be pursued depending on patient and surgeon preference. Surgical decompression provides quick relief and lasting outcomes in the majority of cases. Endoscopic and open techniques are both effective in relieving symptoms and improving motor function.^31^

### Biomechanical analysis

#### Cadaveric evidence of the effects of CTR on the kinematics of carpal bones

Many authors have speculated that the TCL provides stability to the carpal bones. Multiple cadaveric studies have been performed to test this hypothesis and to quantify the degree of stability it provides. The insertion sites of the TCL are thought to play a role in its stabilizing functions. The TCL has a larger area of bony insertion on the trapezium and hook of the hamate compared to the pisiform and scaphoid, and thus likely imparts functionally important stabilization for the trapezium and hamate.^26^^,^^32^

Changes to the carpal arch after CTR have also been demonstrated in several studies.^33^, ^34^, ^35^, ^36^, ^37^ Xiu et al. demonstrated an increase in carpal tunnel outward compliance, or ability to be mobilized in a dorsal direction, of 128% at the distal carpal level and 67% at the proximal carpal levels after TCL sectioning.^36^ Vanhees et al. also demonstrated 30% widening of the carpal arch after sectioning of the TCL.^33^ Moreover, Schiller at el. observed an increase in the carpal arch width specifically between the hamate and the trapezium.^34^ These changes in carpal tunnel width impact the biomechanics of the carpal bones. Widening of the carpal arch, the trapezium, trapezoid, and scaphoid results in an increase in radial deviation of the thumb with axial loading.^33^ Additionally there is 5 degrees of increased scaphoid mobility with just 15 degrees of ulnar deviation. The degree of scaphoid mobility continues to increase with further ulnar deviation.^38^

Rotation of the carpal bones has also been demonstrated. Schiller et al. observed a 4.5 degree rotation in the hamate after CTR and a 2.25 degree rotation in the trapezium.^34^ Furthermore, Gabra et al. examined the total summation of the angular rotations about the pronation/supination axis for the hamate-capitate, capitate-trapezoid, and trapezoid-trapezium joints. They demonstrated an total angular change about the pronation/supination axis of 5.2 degree for a change in carpal arch width of +2 mm and 9.6 degree for a change in carpal arch width of +4 mm.^35^ This summation is comparable to angular change in the axial plane between the hook of hamate and the ridge of the trapezium^35^ which was examined by Flores et al. who reported a 6.2 degree angular change in the axial plane between the hook of hamate and the ridge of the trapezium at TCL sectioning.^39^ Tanner et al. did not find an increase in trapezium rotation although admit their study may be underpowered.^40^ They also examined the distance from the radius to the metacarpal and radius to the trapezium on 5 cadavers both before and after CTR. The summation of the post CTR distances for all specimens was increased compared to pre CTR but was not statistically significant (*p* = 0.1). The authors indicated this may suggest increased laxity and suggested that additional study is needed. The changes in stability of the carpal bones are thought to place increased stress on the intercarpal ligaments. Over time, this stress could then lead to the ligamentous laxity and degeneration that has been implicated as a precipitating factor in CMC arthritis.

Some authors have questioned the clinical implications of an increase in carpal arch width. In cadaver studies, sectioning of the TCL under loading leads to further opening of the palmar arch, but there may be compensatory mechanisms that prevent this instability in vivo.^41^ Additionally, Garcia-Elias et al. demonstrated that a change in carpal tunnel size did not change volar-dorsal stiffness of the transverse intercarpal ligaments, suggesting that they play a crucial role in transverse carpal stability.^37^

Although studies have noticed instability within the carpal arch following CTR, clinical implications remain unclear. Bony and ligamentous intercarpal constraints may be strong enough to permit stable dynamic behavior.^37^^,^^42^ These changes are summarized in Table 1.

**Table 1:** Summary of the anatomic and biomechanical changes following CTR.

Study type	Anatomic change after CTR	Biomechanical implication
Cadaveric		
	Increased carpal arch width and outward compliance	Suggests loss of stability
	Radial deviation of carpal bones	Alters load transmission and thumb mechanics
	Increase in scaphoid mobility	Suggests scaphoid instability
	Increase in rotation of the trapezium and hamate	Contributes to altered CMC biomechanics
Biomechanical		
	Radial and dorsal shift of the scaphoid	May increase inter carpal contact stresses
	Increased triquetral-hamate contact stress	Potential driver of ligament degeneration
	Ulnar and dorsal shift of peak stress location	Alters joint loading patterns
	Increased carpal arch compliance	Suggests loss of stability
Clinical		
	Increased angular changes between the hook of the hamate and the trapezium in open CTR	May contribute to altered CMC biomechanics
	Increased carpal arch width and volume	Suggests in vivo widening similar to cadaveric findings
	Elevated baseline carpal pressure in patients with basal joint arthritis	TCL may be more critical for stability in this population
	Greater widening after open vs endoscopic CTR	Endoscopic CTR may preserve more stability

CTR, carpal tunnel release; CMC, carpometacarpal; TCL, transverse carpal ligament.

#### Clinical evidence of the effects of CTR on the kinematics of carpal bones

Authors have performed clinical, biomechanical studies that have examined in vivo changes to the stability of the carpal bones following CTR. Gartsman et al. reported an average increase of 0.29 cm in the carpal arch width (range, 0.0 to 0.85 cm) – a 13% average increase in the carpal arch width (range, 0% to 52%).^43^ Richman et al. examined the carpal tunnel volume and found a 24.2% ± 11.6% increase in carpal tunnel volume 6 weeks after carpal tunnel release and a 22% ± 13.5% increase in volume at an average of 8 months after operation. This was thought to be secondary to an increase in the convexity of the carpal arch.^44^ Viegas et al. were able to demonstrate changes with endoscopic carpal tunnel release as well. They found endoscopic release increased carpal tunnel width by an average of 1.7 mm (7% increase) from the palmar ridge of the trapezium to the hook of the hamate.^45^ This was the first report to demonstrate that the endoscopic approach yields a smaller degree of widening of the carpal borders than open surgery. Kato et al. also examined endoscopic carpal tunnel release and reported an increase of 33% ± 15% in the postoperative cross-sectional area of the carpal canal, and an increase in the arch width at the level of the hamate from 22.1 to 23.8 mm.^46^ The literature is mixed on outcomes following endoscopic versus open CTR, with several studies demonstrating improved post-operative grip strength and pinch strength with endoscopic release.^47^, ^48^, ^49^ This may be explained by the kinematic changes associated with increased widening of the carpal arch. However, other studies do not demonstrate any long-term differences in functional outcomes.^50^, ^51^, ^52^ To our knowledge there are no studies that specifically examine the relationship between open and endoscopic CTR and CMC arthritis, and further studies evaluating the clinical relevance of this difference in carpal widening and the development of CMC arthritis are needed.

Flores et al. reported increased angular change about the axial plane between the hook of the hamate and the trapezium after TCL sectioning. There were changes in these angles following the division of the TCL after the open and endoscopic procedures; however, statistically significant differences between the pre- and postoperative values were noted only in those cases treated by means of open surgery.^39^ These changes are summarized in Table 1.

#### Computational and biomechanical models of consequences following CTR

Computational and biomechanical models have also demonstrated changes in the bony relationships of the carpus after CTR. Morrell et al. debated the long-term carpal width changes but did corroborate with other studies that carpal tunnel volume increases. This change in carpal arch shape may then stress intercarpal articulations.^53^ Other biomechanical roles have indirectly proven this point by reporting a nine times greater compliance in a released carpal arch.^54^ Guo et al. performed a finite element analysis of the wrist and found the carpal bones deviated more radially after TCL division, the scaphoid specifically moved radially and dorsally with wrist radial deviation and palmar flexion. Contact stress in the triquetrum–hamate joint increased by 29.1%, and the location of the peak stress moved 1.00 mm ulnarly and 0.76 mm dorsally.^55^

Lutsky et al. measured baseline carpal pressure measurements using an intra-compartmental pressure monitor in patients with a radiographic evidence of basal joint arthritis.^56^ They found elevated baseline carpal pressure in their patients compared to data published by Gelberman et al. measuring carpal pressure in patients without basal joint arthritis.^57^ This could indicate the TCL may play a larger role in carpal stability in these patients leading to increased instability after CTR and worsening of the kinematic changes that have been discussed. These changes are summarized in Table 1.

#### Interplay of CTR kinematics and CMC OA

As discussed above CMC OA is thought to occur due to ligamentous laxity and degeneration surrounding the CMC joint. Decreased ligamentous stability of trapezium and thumb metacarpal can lead to greater shear forces across the palmar aspect of the joint. Destabilization of the thumb CMC joint also increases the risk of subluxation. Repeated loading of a subluxed joint may lead to accelerated articular cartilage damage.

Changes in carpal tunnel width and volume of the carpal arch following sectioning of the TCL has been clearly defined. There is also evidence of radial deviation and rotation of the carpal bones - specifically of the hamate and trapezium. These changes have been shown to affect muscle function, grip strength, and stress distribution between the carpal bones. This leads to destabilization of the carpal bones resulting in increased stress on intercarpal ligaments and changes in contact points at intercarpal articulations. When considered together, these alterations may lead to pain due to altered soft tissue forces and/or accelerated articular wear of the thumb CMC joint. A similar hypothesis has been proposed for pisotriquetral pain syndrome where release of the TCL alters pisotriquetral joint alignment and tracking of the pisiform leading to pain.^58^

## Conclusion

There are clearly defined morphologic and biomechanical changes that occur to the carpal arch and carpal bones after CTR. These changes may be associated with differences in intrinsic hand muscle function, increased ligamentous stress, and accelerated ligamentous laxity, leading to alterations in the distribution of force around the trapezium and to its articular cartilage.

## Declaration of competing interest

Authors have no financial disclosure or conflict of interest to report.

## Appendix Supplementary materials

Image, application 1
