# Supplementary material for: A scoping review of the impact of transverse carpal ligament sectioning on the thumb carpometacarpal joint: Does it increase the risk of ostearthritis?
Source: JPRAS Open. 2026 Jan 23;48:980–7. doi: 10.1016/j.jpra.2026.01.029 (PMC12933596; doi:10.1016/j.jpra.2026.01.029)
Supplement: Supplementary file 1 [file mmc1.docx]

**Supplemental Table 1.** Search Terms

| **Search Terms** | |
| --- | --- |
| **PubMed** | Carpometacarpal AND "carpal tunnel release, “carpometacarpal AND carpal tunnel release, carpometacarpal biomechanics AND carp,* Basal AND carpal tunnel release, Basal joint AND carpal tunnel release, Basal joint AND transverse carpal ligament sectioning, Basal joint and transverse carpal ligament sectioning, Basal joint AND transverse carpal ligament, carpometacarpal AND transverse carpal ligament, biomechanics of carpal tunnel release, CMC arthritis AND carpal tunnel release, carpometacarpal arthritis AND carpal tunnel release, basal AND arthritis AND carpal tunnel release, basal AND arthritis AND transverse carpal, transverse carpal ligament release AND trapezium, carpal tunnel AND basal joint, trapeziometacarpal AND carpal, biomechanics AND "carpal tunnel release," biomechanics AND "transverse carpal ligament." |
| **Cochrane Library** | Carpometacarpal AND "carpal tunnel release, “carpometacarpal AND carpal tunnel release, carpometacarpal biomechanics AND carp,* Basal AND carpal tunnel release, Basal joint AND carpal tunnel release, Basal joint AND transverse carpal ligament sectioning, Basal joint and transverse carpal ligament sectioning, Basal joint AND transverse carpal ligament, carpometacarpal AND transverse carpal ligament, biomechanics of carpal tunnel release, CMC arthritis AND carpal tunnel release, carpometacarpal arthritis AND carpal tunnel release, basal AND arthritis AND carpal tunnel release, basal AND arthritis AND transverse carpal, transverse carpal ligament release AND trapezium, carpal tunnel AND basal joint, trapeziometacarpal AND carpal, biomechanics AND "carpal tunnel release," biomechanics AND "transverse carpal ligament." |

**Supplemental Figure 1. Flowchart**

**Identification of studies via databases and registers**

Records removed *before screening*:

Duplicate records removed (n = 764)

Records identified (n=1093)

**Identification**

Records screened

(n = 329)

Records excluded

(n = 244)

Reports sought for retrieval

(n = 85)

Reports not retrieved

(n = 0)

**Screening**

Reports assessed for eligibility

(n = 85)

Reports excluded (n = 37)

Studies included in review

(n = 48)

**Included**
